# Automatic liver segmentation based on appearance and context information

**DOI:** 10.1186/s12938-016-0296-5

**Published:** 2017-01-14

**Authors:** Yongchang Zheng, Danni Ai, Jinrong Mu, Weijian Cong, Xuan Wang, Haitao Zhao, Jian Yang

**Affiliations:** 1 Department of Liver Surgery, Peking Union Medical College Hospital, Chinese Academy of Medical Sciences, Peking Union Medical College, Beijing, 100730 China; 2 Beijing Engineering Research Center of Mixed Reality and Advanced Display, School of Optics and Electronics, Beijing Institute of Technology, Beijing, 100081 China; 3 Department of Radiology, Peking Union Medical College Hospital, Chinese Academy of Medical Sciences, Peking Union Medical College, Beijing, 100730 China

## Abstract

**Background:**

Automated image segmentation has benefits for reducing clinicians’ workload, quicker diagnosis, and a standardization of the diagnosis.

**Methods:**

This study proposes an automatic liver segmentation approach based on appearance and context information. The relationship between neighboring pixels in blocks is utilized to estimate appearance information, which is used for training the first classifier and obtaining the probability distribution map. The map is used for extracting context information, along with appearance features, to train the next classifier. The prior probability distribution map is achieved after iterations and refined through an improved random walk for liver segmentation without user interaction.

**Results:**

The proposed approach is evaluated using CT images with eight contemporary approaches, and it achieves the highest VOE, RVD, ASD, RMSD and MSD. It also achieves a high average score of 76 using the MICCAI-2007 Grand Challenge scoring system.

**Conclusions:**

Experimental results show that the proposed method is superior to eight other state of the art methods.

## Background

Liver segmentation plays a key role in various clinical applications, such as liver diagnosis, tumor segmentation, and liver transplantation [1, 2]. This method faces a challenging problem because of the large variability, irregular shape, low contrast, and tissue adhesion of the live as well as intensified overlapping between organs. At present, liver segmentation can be divided into two categories: image- and statistical model-based methods.

Image-based segmentation uses image brightness, gradient, or texture [3, 4]. Peng et al. [5] proposed a convex variational model for liver semi-automatic segmentation in which gradient, local context-based edge detector, and regional appearance constraint are integrated to enhance the liver detection. Afifi et al. [6] proposed a liver segmentation approach in which the related information obtained between neighboring slices is integrated with the graph cuts algorithm to segment the liver in each CT slice. Chen et al. [7] introduced a strategic combination of the active appearance model, live wire, and graph cuts for liver segmentation. Peng et al. [8] proposed a multiregion-appearance-based approach with graph cuts to delineate liver edge. A geodesic distance-based appearance selection is introduced, and the energy functions incorporating both boundary and region are modeled. Ai et al. [9] proposed a feature-learning-based random walk method for liver segmentation using CT images. Seed points on the original test image were automatically selected.

Statistical model-based segmentation uses a large number of data to establish a general liver location or shape model. Xu et al. [10] developed a 3D-scale invariant feature transform-based registration and designed an objective function to label the target image for liver segmentation. Salman et al. [11] discovered a feature-constrained Mahalanobis distance cost function to determine the active shape model, and liver segmentation is further achieved through a 3D graph cut. He et al. [12] proposed a three-level AdaBoost-guided active shape model for rough segmentation, surface optimization and shape-fitting refinement.

This study proposes an automatic context iteration-based liver segmentation by integrating appearance and statistical information. The gray level co-occurrence matrices (GLCM) are first extracted for appearance representation and used to learn the first classifier. Then, the context features are obtained from a large number of training data with ground truth along with image appearance features to learn the next classifiers. After several iterations, a prior probability liver model is constructed and further segmented through an improved random walk. The main contributions of this work are summarized as follows: (1) the context information rather than only appearance feature is used to construct the prior liver model, and (2) the improved random walk is introduced by integrating both the probability distribution map and original intensity image.

The rest of this paper is organized as follows. After the introduction, the methodology, including prior liver model construction and liver boundary determination, is described in “Methods” section. Evaluation results are presented in “Experimental results” section. “Discussion and conclusion” section concludes the paper.

## Methods

**Fig. 1:**
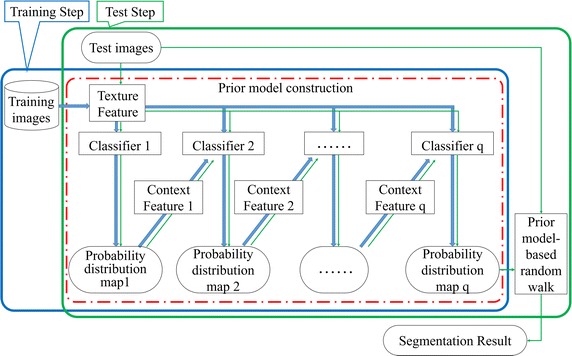
Schematic diagrams for the proposed segmentation method

Motivated in part by context feature extraction and machine learning, we propose an automatic liver segmentation method with two parts and shown in Fig. 1.

1. The prior model construction part contains the training step and test step. The appearance features are first extracted from the test image and training images with label map for classification. Then, the test probability distribution and training probability distribution with label map are obtained to extract the context feature. To achieve the final probability distribution map, both appearance and context features are used for classification on the several iterations.
2. Both intensity and probabilistic information are used by a prior model-based random walk to achieve the final segmentation result.

In this section, the appearance and context-based liver segmentation algorithm is presented in detail. This algorithm is comprised of prior liver model construction and prior model-based random walk.

### Prior liver model construction

Appearance feature is extracted by gray level co-occurrence matrix (GLCM), which reflects the distance and direction between different pixels. In the patch **P** centered on *x*, GLCM calculates probability *p*(*a*, *b*|*d*, *θ*.) wherein the intensity value *a* occurs with intensity value *b* in spatial distance *d* and direction *θ*. Level *Q* (*a*, *b* ∊ *Q*) in an image determines the size of GLCM (*Q* × *Q*). A number of GLCMs are produced for *x* according to different values of *d* and *θ*. Twelve textural features *U* = 12 are used for each GLCM measuring the characteristics of texture statistics. These features are energy, contrast, correlation, homogeneity, entropy, autocorrelation, dissimilarity, cluster shade, cluster tendency, maximum probability [13], statistics variance, and sum mean [14]. The appearance feature for *x* is composed of textural features $f_{u}^{{d_{j} ,\theta_{j} }} ,\left( {u = 1,2, \ldots ,U} \right)$ with given distance *d*_*j*_ and direction *θ*_*j*_(*j* = 1, 2, …*J*)

1 $${\mathbf{f}}_{GLCM} \left( x \right) = \left[ {f_{1}^{{d_{1} ,\theta_{1} }} ,f_{2}^{{d_{1} ,\theta_{1} }} , \ldots ,f_{12}^{{d_{1} ,\theta_{1} }} , \ldots ,f_{12}^{{d_{J} ,\theta_{J} }} } \right]^{T}$$

The training data set is denoted as *V*_*i*_ ∊ *R* ^*N*×*M*^ and the corresponding label map is denoted as *V*_*si*_ ∊ *R* ^*N*×*M*^, *i* = 1, 2, …, *I*, and *I* is the number of training data. Training sample points are expressed with appearance feature ${\mathbf{f}}_{GLCM} \left( {x_{t} } \right)$ and the corresponding label *y*_*t*_

2 $$S_{0} = \left\{ {\left( {y_{t} ,{\mathbf{f}}_{GLCM} \left( {x_{t} } \right)} \right),\quad t = 1,2 \ldots T} \right\} .$$

The first classifier is trained through the AdaBoost algorithm based on the appearance features calculated on local image patch. Probability distribution maps ${\mathbf{P}}_{0} \left( {V_{i} } \right)$ are obtained for the training data set using the learned classifier. We have

3 $$p_{0} \left( {y_{t} = 1|x_{t} } \right) = \frac{{e^{{H_{0} \left( {{\mathbf{f}}_{GLCM} \left( {x_{t} } \right)} \right)}} }}{{e^{{H_{0} \left( {{\mathbf{f}}_{GLCM} \left( {x_{t} } \right)} \right)}} + e^{{ - H_{0} \left( {{\mathbf{f}}_{GLCM} \left( {x_{t} } \right)} \right)}} }}$$

**Fig. 2:**
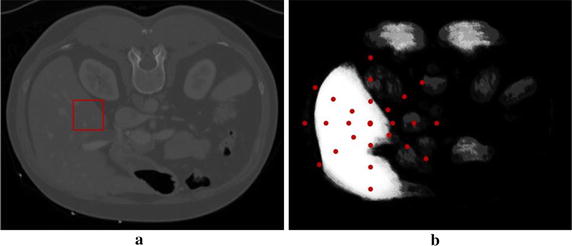
Appearance and context information extracted from the original image and probability distribution map, respectively. **a** Appearance information in the original image; **b** Context information in the probability distribution map

As the largest organ located on the right side of human body, the liver features a special shape and is relatively fixed near other organs. The appearance and context information should be used for liver segmentation simultaneously. Figure 2 shows the appearance and context information extracted from the original image and the probability distribution map, respectively.

In the probability distribution maps, eight rays with 45^∘^ intervals are stretched out from *x*_*t*_, and a radius sequence is used to sample the context locations on each ray. The probabilities on the locations are used as context features ${\mathbf{f}}_{cxt}^{\left( u \right)} \left( {x_{t} } \right)$. Afterward, a series of new training data set is constructed as follows:

4 $${\mathbf{S}}^{\left( u \right)} = \left\{ {\left( {y_{t} ,\left( {{\mathbf{f}}_{GLCM} \left( {x_{t} } \right),{\mathbf{f}}_{cxt}^{\left( u \right)} \left( {x_{t} } \right)} \right)} \right),\quad t = 1,2 \ldots T} \right\} ,$$

where *u* = 1, 2, …, *U* denotes the iteration number. The second classifier based on the appearance and context features $\left( {{\mathbf{f}}_{GLCM} \left( {x_{t} } \right);{\mathbf{f}}_{cxt}^{\left( 1 \right)} \left( {x_{t} } \right)} \right)$ is identified to construct new probability distribution maps ${\mathbf{P}}_{1} \left( {V_{i} } \right)$ in which the classification is expressed as

5 $$p_{1} \left( {y_{t} = 1|x_{t} } \right) = \frac{{e^{{H_{u} \left( {\left( {{\mathbf{f}}_{GLCM} \left( {x_{t} } \right);{\mathbf{f}}_{cxt}^{\left( 1 \right)} \left( {x_{t} } \right)} \right)} \right)}} }}{{e^{{H_{0} \left( {\left( {{\mathbf{f}}_{GLCM} \left( {x_{t} } \right);{\mathbf{f}}_{cxt}^{\left( 1 \right)} \left( {x_{t} } \right)} \right)} \right)}} + e^{{ - H_{0} \left( {\left( {{\mathbf{f}}_{GLCM} \left( {x_{t} } \right);{\mathbf{f}}_{cxt}^{\left( 1 \right)} \left( {x_{t} } \right)} \right)} \right)}} }}$$

**Fig. 3:**
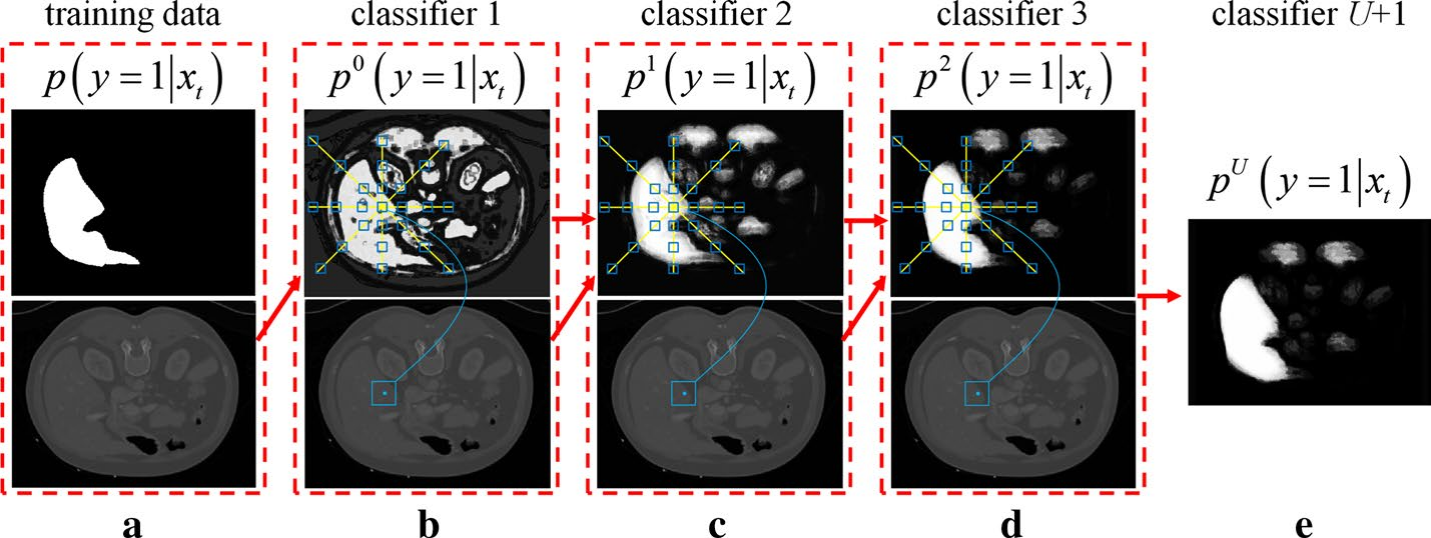
Illustration of the training procedure of prior liver model. Given training data and the corresponding segmented liver (**a**), the first classifier is learned based on appearance features and the initial probability distribution map (**b**) is obtained. Then the appearance and context features are combined to learn a subsequent classifiers (**c**, **d**). Here, the context features are extracted from the probability distribution map produced by the previous classifier. After *U* + 1 iterations, the final probability distribution map ${\mathbf{P}}_{U} \left( {x_{t} } \right)$ is realized (**e**)

A similar learning procedure is performed to obtain *U* learned classifiers. The iteration procedure is shown in Fig. 3. Figure 3a shows the training data *V*_*i*_ (bottom line) and the corresponding segmented liver *V*_*is*_ (top line). Based on the appearance features extracted from the original image, the initial classification, denoted as *p* ^0^(*y*_*t*_ = 1|*x*_*t*_.), provides the probability distribution map ${\mathbf{P}}_{0} \left( {V_{i} } \right)$ shown in Fig. 3b (top line). The appearance and context features are then integrated and processed through multiple iterative classification (Fig. 3c, d) to obtain the final classification result *p* ^*U*^(*y*_*t*_ = 1|*x*_*t*_.), which is shown in Fig. 3e as the probability distribution map ${\mathbf{P}}_{U} \left( {V_{i} } \right)$.

For the test data *V*, the prior liver model *V*_*s*_ is obtained through the sequence of classifiers based on both appearance and context features. The same procedure performed on the training data is implemented, including appearance feature extraction, context feature extraction, classifier learning and iteration loop until convergence. The final classification result for each pixel constructs the probability distribution map ${\mathbf{P}}_{U} \left( V \right)$.

### Prior model-based random walk

The original text image and probability distribution map are defined as two corresponding graphs. Nodes *v*_*i*_ are pixels, and edges *e*_*ij*_ are produced between two neighbor pixels. The original random walk [15] has two limitations: (1) the procedure is not automatic such that the seeds have to be manually appointed by the user and (2) only the intensity information is not distinguishable for liver segmentation. To address these problems, prior liver model is used for seed determination [16], that is, pixels *x*_*t*_ with $p(y_{t} = 1\left| {v_{t} } \right.){ = 1}$ are denoted as liver seeds, and pixels *x*_*t*_ with $p(y_{t} = 1\left| {v_{t} } \right.){ = 0}$ are denoted as background seeds. Both the intensity and probability distribution information are utilized to calculate the most significant parameter of random walk, that is, the weight *w*_*ij*_ of *e*_*ij*_:

6 $$\omega_{ij} = \exp \left( { - \beta \left( {\left( {1 - \alpha } \right)\left( {G\left( {v_{i} } \right) - G\left( {v_{j} } \right)} \right)^{2} + \alpha \left( {P(v_{i} ) - P(v_{j} )} \right)^{2} } \right)} \right)$$

where *G*(*v*_*i*_) and *P*(*v*_*i*_) are the pixel intensity value and corresponding probability distribution value of the original image and probability distribution map, respectively, and *α* and *β* are the adjusting parameters. When liver pixel *v*_*i*_ and background *v*_*j*_ are near the liver edge with similar intensity values but different probabilities, relatively precise segmentation result can be achieved.

According to the original random walk, the final probability of *v*_*i*_ is determined by the minimum objective function as follows

7 $$E_{spatial}^{s} = x^{sT} Lx^{s} = \sum\limits_{{}} {\omega_{ij} (v_{i}^{s} - v_{j}^{s} )}^{2} ,\quad s.t. \, L_{{v_{i} v_{j} }} = \left\{ {\begin{array}{*{20}c} {d_{{v_{i} }} } \\ { - \omega_{ij} } \\ 0 \\ \end{array} } \right. \, \begin{array}{*{20}c} {i = j} \\ {v_{i} ,v_{j} {\text{are neighbors}}} \\ {\text{others}} \\ \end{array}$$

where *x*_*i*_^*s*^ is the probability of each pixel in the image belonging to the different classes, and *s* denotes the liver *s* = 1 or background *s* = 0. After finding the value of *x*_*i*_^*s*^, we can determine to which class a certain pixel belongs:

8 $$label(v_{i} ) = \left\{ {\begin{array}{l} {1, \quad x_{i}^{s} \ge 1/2 \, } \\ {0, \quad x_{i}^{s} < 1/2} \\ \end{array} } \right.$$

## Experimental results

In this section, the proposed method is evaluated on the MICCAI 2007 grand challenge data [17], which contains 20 3D abdominal CT scans with ground truth. Pixel spacing varies from 0.55 to 0.8 mm in x/y-direction, and slice distance varies from 1 to 3 mm [18]. Tumors, metastasis, and cysts with various sizes are included in most images. Transversal directions were acquired for CT scans with segmented livers. The number of slices in each scan varies between 64 and 394 with 512 × 512 resolution.

Rather than the whole CT scan, only a single slice that contains the largest liver is used as training data. Two scans randomly select in MICCAI 2007 grand challenge database serve as the test data. Eighteen slices contained in the remaining 18 scans are used as training data.

### Objective evaluation

Five evaluations are implemented for assessing how the results of the proposed method *B* will generalize to the ground truth *A*. The five evaluation measurements are generally defined as follows [19].

Volume overlap (VOE):

9 $$VOE = 1 - \frac{{vol\left( {A \cap B} \right)}}{{vol\left( {A \cup B} \right)}}$$

Relative volume difference (RVD):

10 $$RVD = \frac{{vol\left( {A\backslash B} \right)}}{vol\left( B \right)}$$

Average surface distance (ASD):

11 $$ASD(A,B) = {{\left( {\sum\limits_{{s_{A} \in S(A)}} {d(s_{A} ,S(B)) + \sum\limits_{{s_{B} \in S(B)}} {d(s_{B} ,S(A))} } } \right)} \mathord{\left/ {\vphantom {{\left( {\sum\limits_{{s_{A} \in S(A)}} {d(s_{A} ,S(B)) + \sum\limits_{{s_{B} \in S(B)}} {d(s_{B} ,S(A))} } } \right)} {(|S(A)| + |S(B)|)}}} \right. \kern-0pt} {(|S(A)| + |S(B)|)}}$$

Root mean squared error (RMSD):

12 $$RMSE(A,B) = {{\sqrt {\left( {\sum\limits_{{s_{A} \in S(A)}} {d^{2} (s_{A} ,S(B)) + \sum\limits_{{s_{B} \in S(B)}} {d^{2} (s_{B} ,S(A))} } } \right)} } \mathord{\left/ {\vphantom {{\sqrt {\left( {\sum\limits_{{s_{A} \in S(A)}} {d^{2} (s_{A} ,S(B)) + \sum\limits_{{s_{B} \in S(B)}} {d^{2} (s_{B} ,S(A))} } } \right)} } {\sqrt {(|S(A)| + |S(B)|)} }}} \right. \kern-0pt} {\sqrt {(|S(A)| + |S(B)|)} }}$$

Maximum surface distance (MSD):

13 $$MSD(A,B) = \hbox{max} \left\{ {\mathop {\hbox{max} }\limits_{{s_{A} \in S(A)}} d^{2} (s_{A} ,S(B)),\mathop {\hbox{max} }\limits_{{s_{B} \in S(B)}} d^{2} (s_{B} ,S(A))} \right\}$$

where *vol*(*) denotes the volume of the region *, *S*(*) is the surface voxel of the region *, s_*_ is one of the voxels on the surface of the region *, and $d(s_{{ *_{1} }} ,S( *_{2} )) = \mathop {\hbox{min} }\limits_{{s_{{ *_{2} }} \in S( *_{2} )}} ||s_{{ *_{1} }} - s_{{ *_{2} }} ||$ is the minimum Euclidean distance between corresponding voxels of two data surfaces. According to the above definition, VOE and RVD give the whole comparison for two segmented livers match, while ASD, RMSE and MSD indicate the difference of the pixel surface distance.

### Context iteration-based classification results

**Fig. 4:**
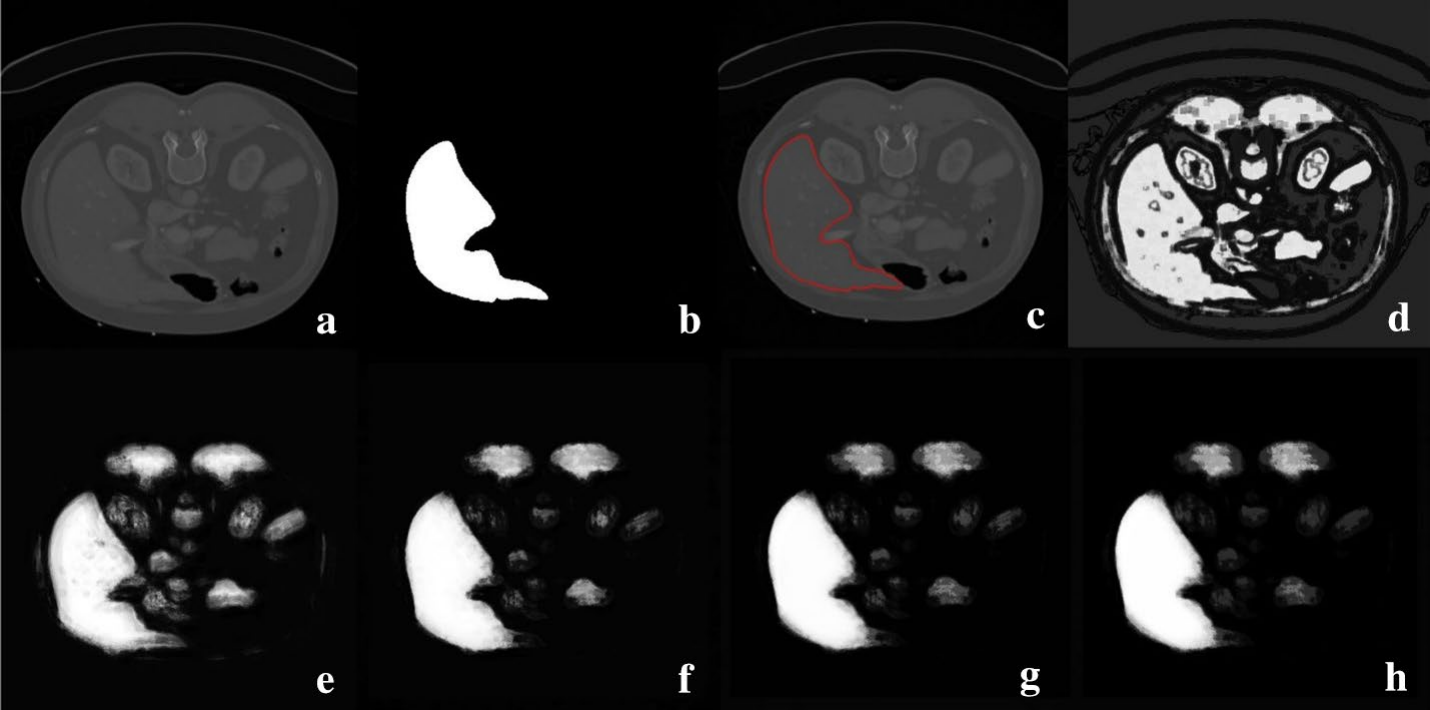
Context iteration-based classification. **a** The original image; **b** the ground truth; **c** the contour profile of the liver; **d** GLCM-based classification result; **e**–**h** classification result based on four iterations

To detect the validity of context iteration, two kinds of classifications are illustrated. One classification is obtained with GLCM features, and the other is achieved with both GLCM and context iteration. In Fig. 4d, the liver can not be distinguished from muscles and other organs when only GLCM features are utilized. Context iteration considers both texture and context information, as shown in Fig. 4e–h. The significance of the liver is substantially improved, whereas the non-liver areas are weakened by adding the context feature to the texture feature.

**Fig. 5:**
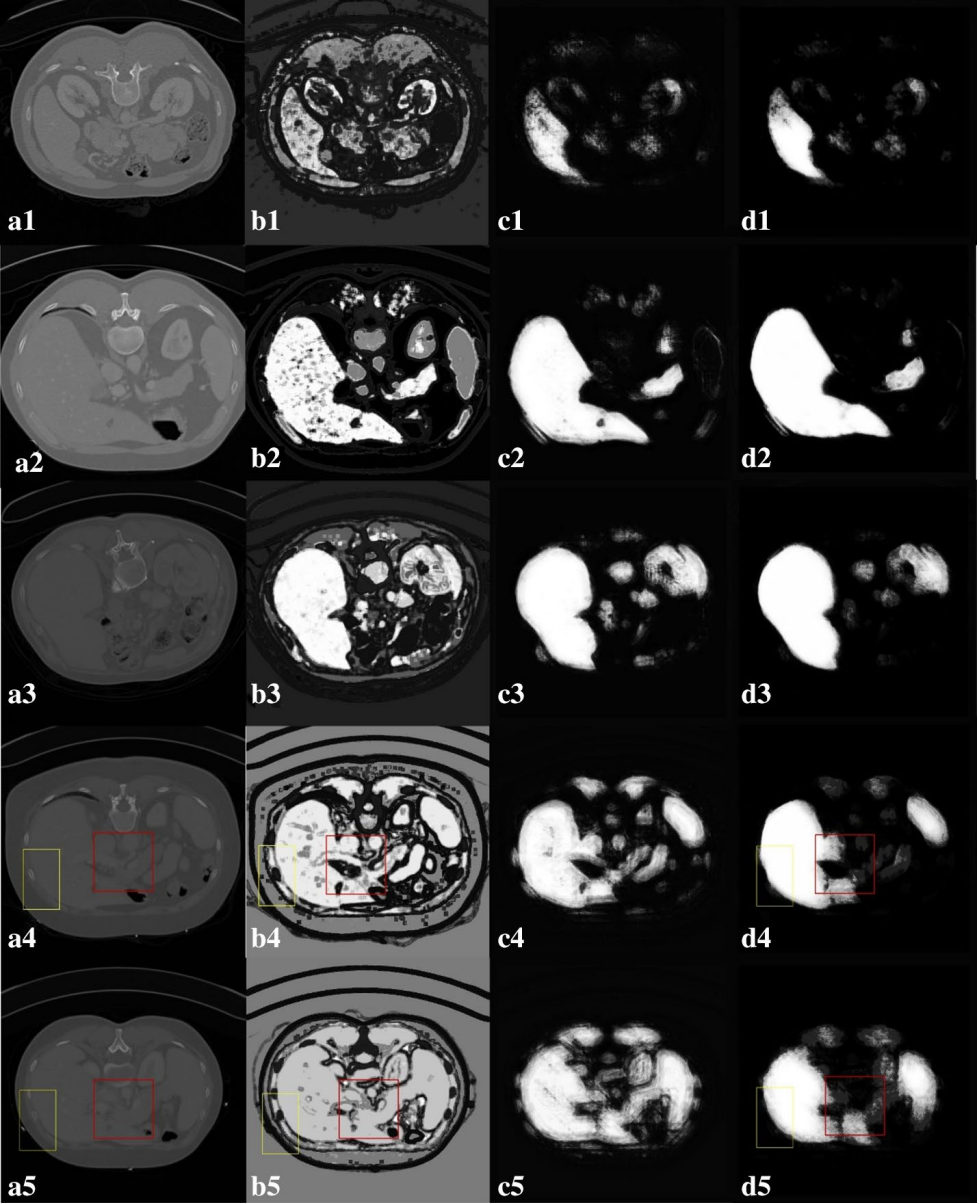
Context iteration-based classification of five slices. **a** Original image; **b** GLCM-based classification result; **c** context iteration-based classification result for the first; **d** the five iteration

The context iteration-based classifications of five slices are shown in Fig. 5. Figure 5a shows the original image. GLCM-based classification results are shown in Fig. 5b. The context iteration-based classification results for the first and fifth iteration are shown in Fig. 5c and d, respectively. Compared with the GLCM-based classification results shown in Fig. 5b1–b3, the probability difference between the liver and the background is large and the significance of the liver is substantially improved based on the five context iteration, as shown in Fig. 5d1–d3. The red and yellow boxes in Fig. 5a4, a5 indicate similar intensity between the liver and the kidney or vessels (red box), and between the liver and muscle (yellow box). The liver cannot be segmented using only GLCM features, as shown in Fig. 5b4, b5. After considering context information, the contrasts between the liver and the other organs are apparent as shown in the red and yellow boxes in Fig. 5d4, d5. The prior probability model effectively provides the shape information of the liver.

### Experimental error analysis

**Fig. 6:**
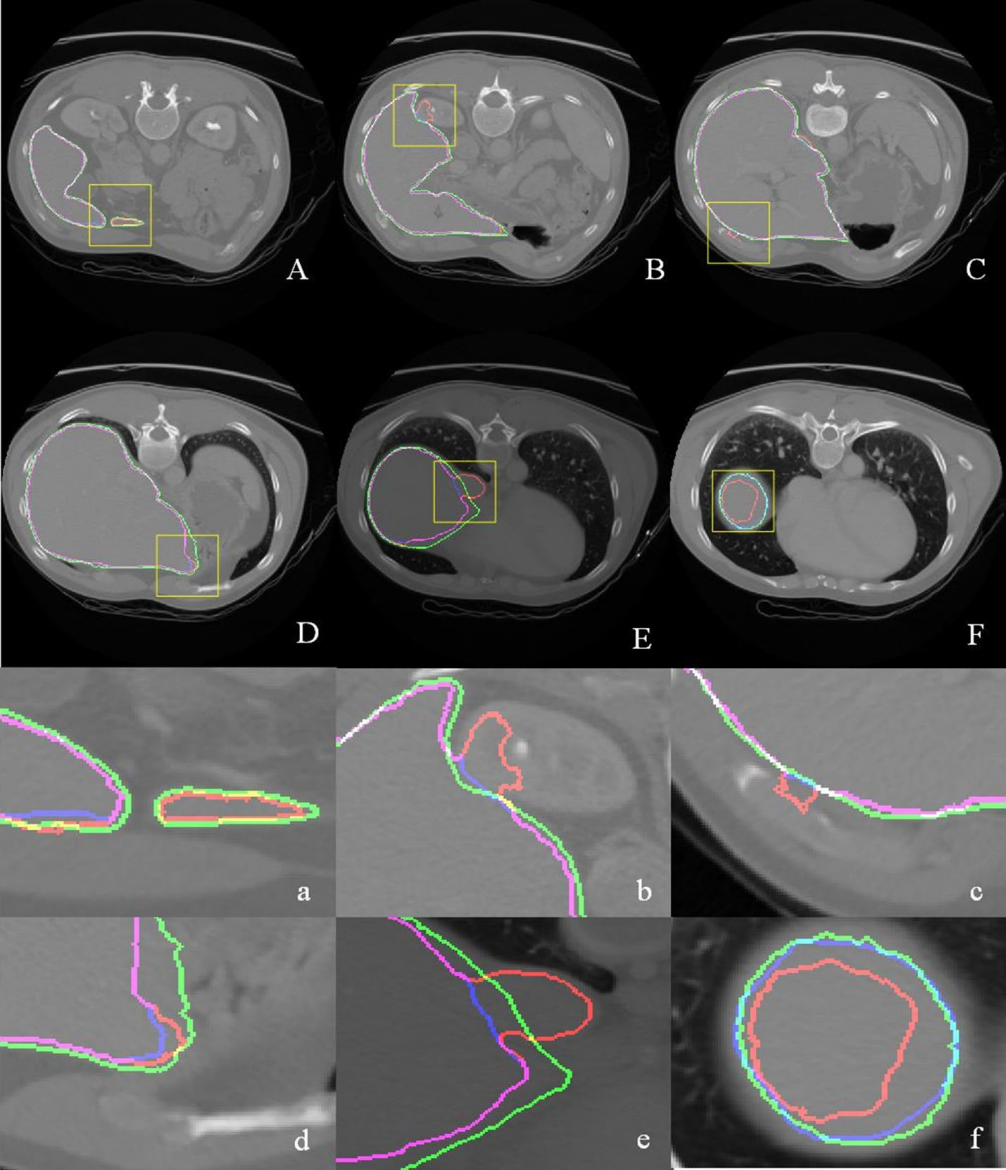
Comparison of the results among the ground truth (*green curves*), GLCM-based classification (*red curves*) and context iteration-based classification (*blue curves*). The segmentation results in six slices are displayed in **A**–**E**, and the corresponding enlarged parts in the *yellow boxes* are shown in **a**–**e**

The segmentation results in six slices are displayed in the first two lines of Fig. 6. Three segmentation results are compared using different colors, particularly, the ground truth (green curves), GLCM-based segmentation (red curves), and context iteration-based classification (blue curves). The contents in the yellow boxes are enlarged to illustrate the details, as shown in the last two lines of Fig. 6. The GLCM-based classification evidently resulted in over-segmented or under-segmented results because of the similar intensities between the livers and background. Context iteration-based classification can provide more precise segmentation than the GLCM-based classification. However, context iteration-based classification is inadequate in the corner, as shown in Fig. 6d, e. This finding is caused by the decrease in prior probability. The proposed method improves the segmentation results for the conjunction of the liver and kidney/muscle/vessels.

**Fig. 7:**
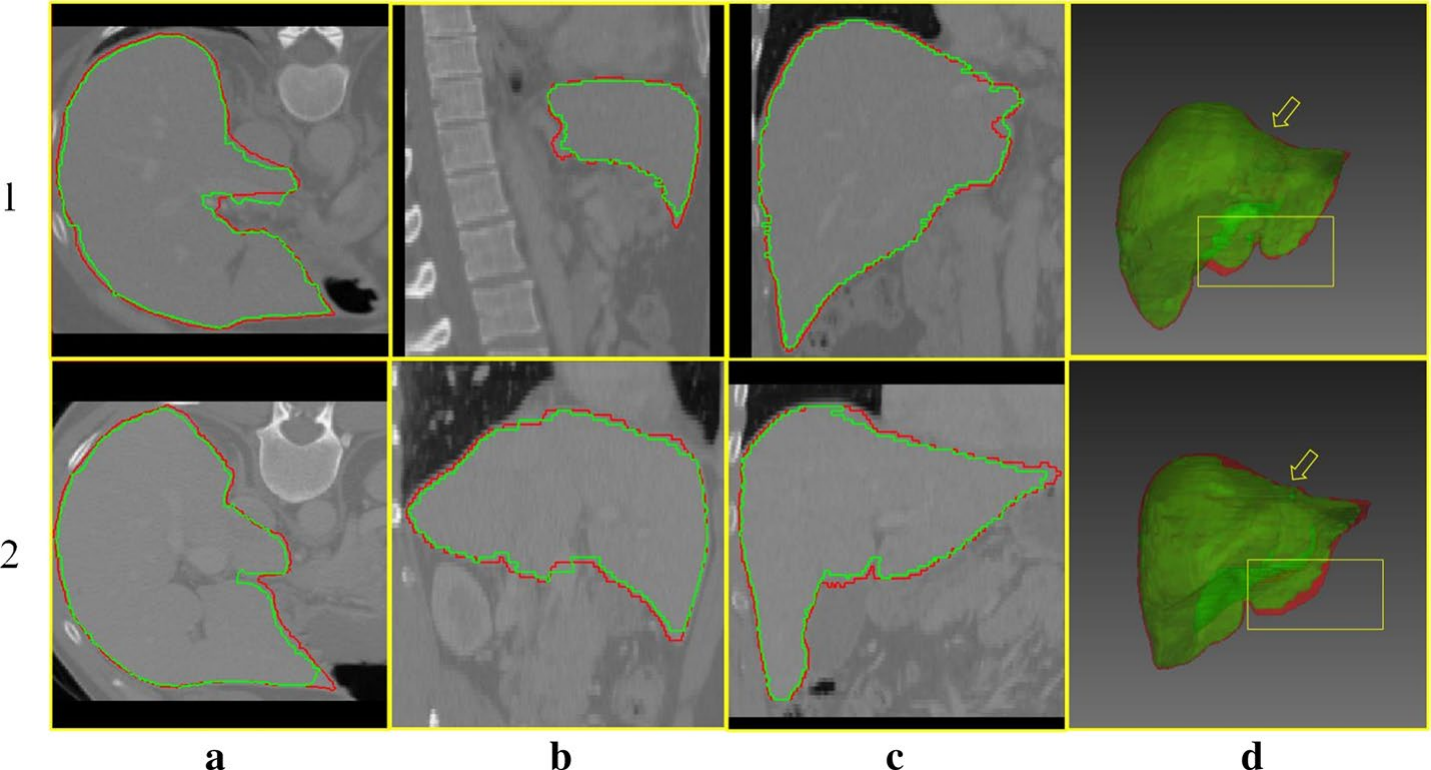
Two-dimensional segmentation results on different directions and 3D segmentation fusion. The first and second rows show two segmentation results of the test data. The coronal, vertical, and transverse sections of two data are shown in **a**–**c** columns. The 3D segmentations are shown in **d** column

Two different segmentation results of the test data are shown in Fig. 7. Figure 7a–c illustrate the 2D segmentation results on the coronal, vertical, and transverse sections. The red contour profile indicates the ground truth, and the green contour profile indicates the context-based iteration segmentation. The segmentation results accurately fit the liver edge, even in the concave region of the liver. The 3D segmentations of the ground truth and proposed methods are shown in Fig. 7d. Precise segmentation is obtained in the area indicated by the yellow arrow. Under-segmentation is produced in the area of the yellow box and should be improved in our future work.

Five evaluations, namely, VOE, RVD, ASD, RMSD and MSD, are implemented for assessing nine automatic segmentation methods (Table 1). The proposed method achieves 7.83% VOE, 5.06% RVD, 1.06 mm ASD, 1.39 mm RMSD, 11.12 mm MSDD, 1.39 mm RMSD, and 11.12 mm MSD. The corresponding score for test data is obtained by [18]. The comparative results show that the proposed method is superior to eight other methods with regard to the total score and exhibits a good applicability for liver segmentation. The total score of the proposed method is 76, which is higher than the standard score provided by [18]. This result indicates that the proposed method satisfies the requirement of clinical liver segmentation.

**Table 1:** The score comparisons of different automatic segmentation methods with MICCAI database

Methods	VOE		RVD		ASD		RMSE		MSD		Total
	[%]	Score	[%]	Score	[mm]	Score	[mm]	Score	[mm]	Score	Score
Ours	7.83	69	5.06	73	1.06	74	1.39	80	11.12	85	76
Heimann [20]	7.77	70	1.7	88	1.4	65	3.2	55	30.1	60	67
Saddi [21]	8.9	65	1.2	80	1.5	62	3.4	52	29.3	62	64
Schmidt [22]	10.4	59	4.9	74	1.7	58	3.1	57	24	68	63
Chi [23]	9.1	65	26	73	1.7	58	3.3	54	30.8	60	62
Rusko [24]	10.1	61	3.8	72	1.7	58	3.5	53	26.7	65	61
Seghers [25]	10.7	58	6.8	64	1.8	55	3.2	56	25.2	67	60
Furukawa [26]	10.8	58	7.3	61	1.9	53	3.7	49	31.6	58	56
van Rikxoort [27]	12.5	51	1.8	80	2.4	40	4.4	40	32.4	57	53

## Discussion and conclusion

In this paper, we developed machine learning techniques for the automatic liver segmentation in CT images. Appearance and context features are extracted for classifying image pixels based on AdaBoost algorithm. This classification provides the prior probability distribution map, which is combined with the original image to segment the liver through the improved random walk. Five evaluations, namely, VOE, RVD, ASD, RMSD and MSD, are implemented for assessing the efficiency of the proposed segmentation method in MICCAI 2007 grand challenge database with 20 abdominal CT images. Experimental results show that the proposed method achieves significantly more accurate results than the other eight state-of-art segmentation methods by comparing VOE, RVD, ASD, RMSD and MSD measurements. In addition, the total score of the proposed method is also higher than that of the ground truth which is manually labeled by medical experts.

The context features take full advantage of the context and high-level information. The intra-object configuration, and inter-object relationship are both considered to distinguish the organs with similar intensity. The saliency of liver is significantly improved compared to other organs.

However, several limitations of the proposed method still exist and should be improved in future work: (1) only an appearance is connected with context feature, which is somewhat limited and requires more explicit shape information; (2) after more features concatenated for expressing, the feature fusion method should be utilized to reduce the redundancy of features (3) the running time takes a long for the image analysis, because all pixels containing in the image have to be analyzed on the serial calculation. GPU accelerated algorithm should be developed in our future work.

## Abbreviations

GLCM: gray level co-occurrence matrix
VOE: volume overlap
RVD: relative volume difference
ASD: average surface distance
RMSD: root mean squared error
MSD: maximum surface distance
